# Audiometric Characteristics of Hyperacusis Patients

**DOI:** 10.3389/fneur.2015.00105

**Published:** 2015-05-15

**Authors:** Jacqueline Sheldrake, Peter U. Diehl, Roland Schaette

**Affiliations:** ^1^The Tinnitus and Hyperacusis Centre, London, UK; ^2^Institute of Neuroinformatics, ETH and University of Zurich, Zurich, Switzerland; ^3^UCL Ear Institute, London, UK

**Keywords:** hyperacusis, tinnitus, loudness discomfort levels, audiogram, hearing loss

## Abstract

Hyperacusis is a frequent auditory disorder where sounds of normal volume are perceived as too loud or even painfully loud. There is a high degree of co-morbidity between hyperacusis and tinnitus, most hyperacusis patients also have tinnitus, but only about 30–40% of tinnitus patients also show symptoms of hyperacusis. In order to elucidate the mechanisms of hyperacusis, detailed measurements of loudness discomfort levels (LDLs) across the hearing range would be desirable. However, previous studies have only reported LDLs for a restricted frequency range, e.g., from 0.5 to 4 kHz or from 1 to 8 kHz. We have measured audiograms and LDLs in 381 patients with a primary complaint of hyperacusis for the full standard audiometric frequency range from 0.125 to 8 kHz. On average, patients had mild high-frequency hearing loss, but more than a third of the tested ears had normal hearing thresholds (HTs), i.e., ≤20 dB HL. LDLs were found to be significantly decreased compared to a normal-hearing reference group, with average values around 85 dB HL across the frequency range. However, receiver operating characteristic analysis showed that LDL measurements are neither sensitive nor specific enough to serve as a single test for hyperacusis. There was a moderate positive correlation between HTs and LDLs (*r* = 0.36), i.e., LDLs tended to be higher at frequencies where hearing loss was present, suggesting that hyperacusis is unlikely to be caused by HT increase, in contrast to tinnitus for which hearing loss is a main trigger. Moreover, our finding that LDLs are decreased across the full range of audiometric frequencies, regardless of the pattern or degree of hearing loss, indicates that hyperacusis might be due to a generalized increase in auditory gain. Tinnitus on the other hand is thought to be caused by neuroplastic changes in a restricted frequency range, suggesting that tinnitus and hyperacusis might not share a common mechanism.

## Introduction

Hyperacusis is an auditory disorder that is characterized by an “unusual tolerance for everyday sounds” (1), an “abnormal reduced tolerance to environmental sound” (2), or “abnormal increased sound-induced activity within the auditory pathways” (3). Many patients describe that everyday sounds, i.e., sounds that would generally be considered to be of normal loudness and comfortable to listen to, are too loud or unbearably loud, causing them discomfort or even pain. Other forms of decreased sound tolerance are misophonia (strong dislike of sounds) or phonophobia (fear of sounds), where specific sounds cause aversive reactions regardless of sound intensity (3). In hyperacusis, on the other hand, problems are generally related to sound intensity, and not restricted to specific types of sounds (3, 4).

Hyperacusis can have a strong impact on the quality of life, as it often leads to changes in behavior like avoiding loud situations, social interactions, public transport, all of which impede the patients’ ability to lead a normal life. For the prevalence of hyperacusis, a certain range has been reported in the literature, e.g., 2% (5), 8.6% (6), or even 15.2% (7). However, even the most conservative estimate of 2% indicates that this is a quite frequent disorder that affects millions.

Hyperacusis shows a high degree of co-morbidity with the phantom auditory sensation of tinnitus. It is estimated that 86% of hyperacusis patients also perceive tinnitus (4). However, only around 27–40% (3, 8, 9) of people with tinnitus also report hyperacusis symptoms, but a higher prevalence of 79% has also been reported (10). Note, however that the latter study was based on a much smaller sample than the former. Moreover, tinnitus subjects with normal hearing thresholds (HTs) have been reported to exhibit decreased LDLs and increased loudness growth, whereas tinnitus subjects with hearing loss did not show such signs of hyperacusis on average (11). It has thus been speculated that tinnitus and hyperacusis might have a shared etiology or might be due to the same pathological mechanism, for example, increased gain in the auditory system.

Since hyperacusis is characterized by abnormal loudness perception, measurements of loudness discomfort levels (LDLs) and loudness growth have been used to study hyperacusis. Anari et al. (4) studied 100 patients with hyperacusis. Most patients had normal or near-normal HTs. LDLs were measured at 0.5, 1, 2, 3, and 4 kHz, and were similar across frequencies, averaging between 75 and 80 dB HL, thus showing a decrease compared to normal values, which are in the order of 100–105 dB HL (12). A similar decrease of LDLs in subjects with hyperacusis has been reported by Formby et al. (13) for LDLs measured at 1, 2, 4, and 8 kHz. So far, LDLs at frequencies below 0.5 kHz have not been reported, and no study has investigated the full range of audiometric frequencies.

Loudness growth in subjects with hyperacusis has been studied by Brandy and Lynn (14) and Norena and Chery-Croze (15). Brandy and Lynn measured loudness growth for 1 kHz tones in 25 subjects with hyperacusis. Compared to the control group, they exhibited both steeper growth of perceived loudness and a lower value for loudness discomfort. Norena and Chery-Croze investigated loudness growth at three different frequencies that were chosen for each participant based on their audiogram. All participants had high-frequency hearing loss, and thus one frequency was chosen to be in the region of hearing loss, one at the audiogram edge, and one at low frequencies where hearing was normal or near-normal. The average pattern for the eight study participants was that loudness growth was abnormally steep at all three frequencies. Interestingly, the discomfort level was roughly the same for all three frequencies, even though the HTs differed considerably. Taken together, these findings indicate abnormal processing of sounds in hyperacusis, and possibly a certain frequency-independent general discomfort level. However, frequencies below 0.5 and above 4 kHz have not yet been systematically investigated, and thus it has remained unclear whether the discomfort levels really show such a pattern.

Here, we report the HTs and LDLs of a group of 381 patients with a primary complaint of hyperacusis. Both HTs and LDLs were measured from 125 to 8 kHz. Moreover, we also compared patient LDL results to a reference population to investigate sensitivity and specificity of LDLs as a measure of hyperacusis.

## Materials and Methods

### Patients

This study was a retrospective analysis of anonymized data that had been routinely collected from patients that attended the London Tinnitus and Hyperacusis Centre between 1979 and 2012. Three hundred eighty-one patients (170 female and 211 male) with a primary complaint of hyperacusis were identified in the database. All patients underwent audiometry and LDL testing at the intake examination. The diagnosis of hyperacusis was established based on patient history and description of symptoms. The average age of the female subjects was 47.2 ± 15.7 years, the average age of the male subjects was 40.8 ± 13.7 years, which gives an overall average age of 43.9 ± 15.0 years.

### Audiometry

All measurements were conducted in a sound-proof booth using a calibrated clinical audiometer (Kamplex KC 30) with Telephonics TDH 39 headphones. All audiometric testing was done by a single person (Jacqueline Sheldrake) following the same protocol for all patients. The HTs and the LDLs of the subjects were measured at 0.125, 0.25, 0.5, 1, 2, 4, 6, and 8 kHz. LDLs were measured by presenting 0.5 s long pure tones of increasing level (5 dB steps), and patients were asked to indicate when they did not want to be presented with the next sound. The level at which the test was stopped was then taken as the LDL. If the LDL was not reached up to the maximum output level of the audiometer (90, 110, 120, 120, 120, 120, 120, and 100 dB HL for 0.125, 0.25, 0.5, 1, 2, 4, 6, and 8 kHz, respectively), we substituted the corresponding LDL by the maximum output level plus 5 dB.

### Data analysis and statistical test

All patient data was stored in a database and then imported into SciPy and Matlab for further analysis, e.g., calculation of means, medians, SDs, construction of histograms, and cumulative distribution functions. To analyze distributions of LDLs and HTs of each ear, the average value of each measure was computed for each ear for the frequency range of 0.5–6 kHz. This restricted frequency range was chosen since the maximum output of our audiometer was constant (120 dB HL) in this range. We then computed histograms from these average values.

Correlations were analyzed using the Pearson correlation coefficient, which is calculated using

$$r=\frac{\sum_{i=1}^{n}\left(x_{i}-\bar{x}\right)\left(y_{i}-\bar{y}\right)}{\sqrt{\sum_{i=1}^{n}{\left(x_{i}-\bar{x}\right)}^{2}}\sqrt{\sum_{i=1}^{n}{\left(y_{i}-\bar{y}\right)}^{2}}}$$

where *n* is the number of examples, *x* and *y* are the tested quantities, and $\bar{x}$ and $\bar{y}$ are the corresponding means $\bar{x}=\frac{1}{n}\sum_{i=1}^{n}x_{i}.$ The correlation coefficient ranges from −1 to 1, where −1 indicates perfect anti-correlation and 1 perfect correlation.

Receiver operating characteristic (ROC) curves were constructed to visualize sensitivity and specificity of LDLs as diagnostic tools for hyperacusis. ROC curves are a common tool to visualize the rate of true and false positives of a test for all possible values of the discrimination threshold. Here, we have based discrimination on LDL values, with values up to the threshold categorized as hyperacusis, and higher values classified as normal. To construct the ROC curves, we thus determined for each LDL threshold value (from 0 to 120 dB HL) how many percent of the patients (true positives) and the reference group (false positives) had an LDL lower than or equal to the threshold. Thus, along the ROC curves, the threshold increases, and the trade-off between detection (true positives) and false alarms (false positives) can be seen. Specificity is then simply given by 1 – false positives.

### Comparison to normative LDL values

Normative LDL data were obtained from graphs published in Ref. (12).

## Results

**Figure 1:**
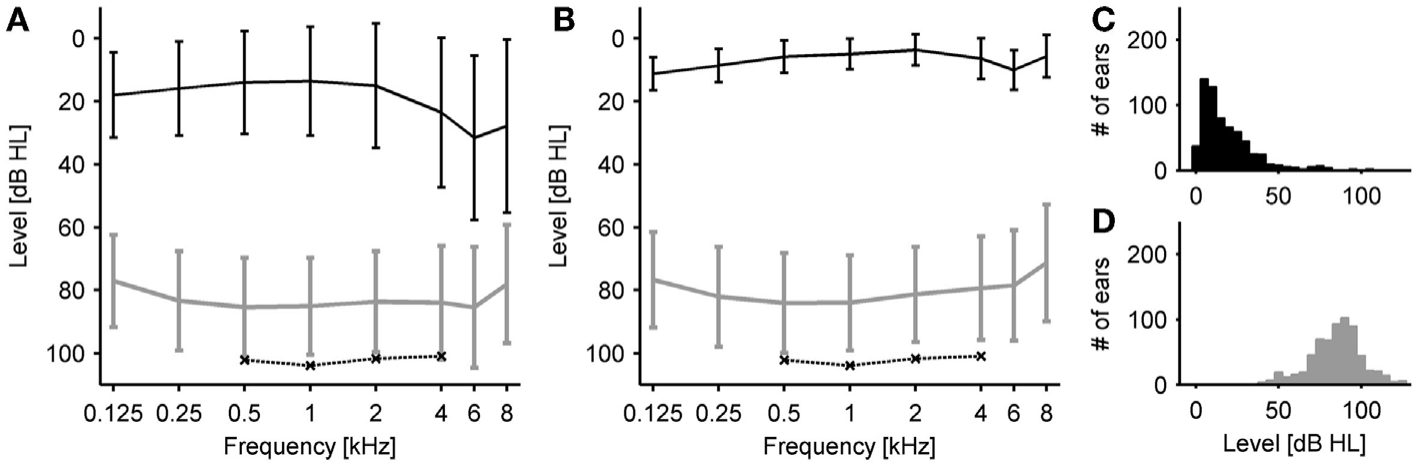
**Hearing thresholds and loudness discomfort levels (LDLs)**. **(A)** Average hearing thresholds (black) and LDLs (gray) of all patients. Error bars denote ± 1 SD. Error bars denote ±1 SD. The dashed line indicates LDLs of a reference group with normal hearing thresholds from Sherlock and Formby (12). **(B)** Average hearing thresholds (black) and LDLs (gray) of a subgroup of patients with clinically normal hearing thresholds. Error bars denote ±1 SD. **(C)** Distribution of hearing thresholds. For each ear, the average hearing threshold was calculated for the frequency range of 0.5–6 kHz. **(D)** Distribution of LDLs. For each ear, the average LDL was calculated for the frequency range of 0.5–6 kHz.

We measured and analyzed HTs and LDLs of 381 hyperacusis patients. Eighty-six percent of the patients also reported tinnitus. On average, the patients had normal HTs (i.e., ≤20 dB HL) at low frequencies and mild hearing loss in the high frequency range (HTs 20–40 dB HL at 4–8 kHz), see Figure 1A. In contrast, the average LDLs were almost constant across the frequency range, with values around 85 dB HL (range 78–87 dB HL). Compared to the normative estimates of Sherlock and Formby (12), mean LDLs of our patient group were thus decreased by 15.7–17.8 dB for 0.5–4 kHz (see also Table 1). Almost the same LDL pattern, with slightly lower LDLs, was observed when we only analyzed ears with normal HTs, i.e., HTs ≤20 dB HL from 125 to 8 kHz (Figure 1B; 37% of all tested ears). Finally, we averaged HTs and LDLs for each ear across frequencies from 0.5 to 6 kHz (since the output limit of the audiometer was 120 dB HL for all these frequencies), to derive the distributions of mean HTs (Figure 1C) and mean LDLs (Figure 1D).

**Table 1:** **Average LDLs of all patients, of the subgroup of patients with normal hearing thresholds, and normative estimates for LDLs from a study by Sherlock and Formby (12)**.

	125 Hz	250 Hz	500 Hz	1 kHz	2 kHz	4 kHz	6 kHz	8 kHz
Patient LDLs (dB HL)	77.0	83.4	85.4	85.1	83.7	84.0	85.4	78.0
SD (dB)	14.6	15.7	15.7	15.3	16.0	18.1	19.2	18.8
NH patient LDLs (dB HL)	76.7	82.1	84.1	84.0	81.3	79.4	78.5	71.3
SD (dB)	15.2	15.8	15.9	15.1	15.1	16.4	17.5	18.6
Normative LDLs (dB HL)			102.2	103.9	101.7	100.9		
SD (dB)			11.8	10.7	12.0	13.6		

**Figure 2:**
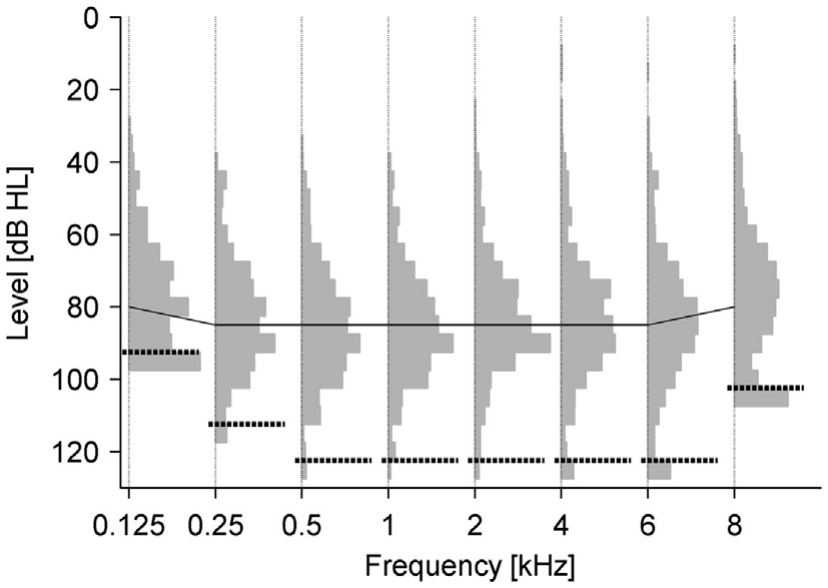
**Distribution of LDLs for individual frequencies**. LDL distributions are depicted by gray bars, results where the LDL could not be reached until the intensity limit of the audiometer are separated by dashed black lines. The solid black line denotes the mean LDL.

Since the range of LDL values was surprisingly large, we also analyzed the distribution of LDLs at all frequencies. The lowest values were around 30 dB HL, only very few results patients indicated discomfort at even lower levels (Figure 2). Surprisingly, for a small fraction of the patients, the LDL could not be reached up to the intensity limit of the audiometer (Figure 2). However, this “problem” was most pronounced at 125 Hz and 8 kHz, where the audiometer only reached 90 and 100 dB HL, respectively. The distributions had a remarkably similar shape at all frequencies, indicating again that hyperacusis symptoms might not be frequency-specific.

**Figure 3:**
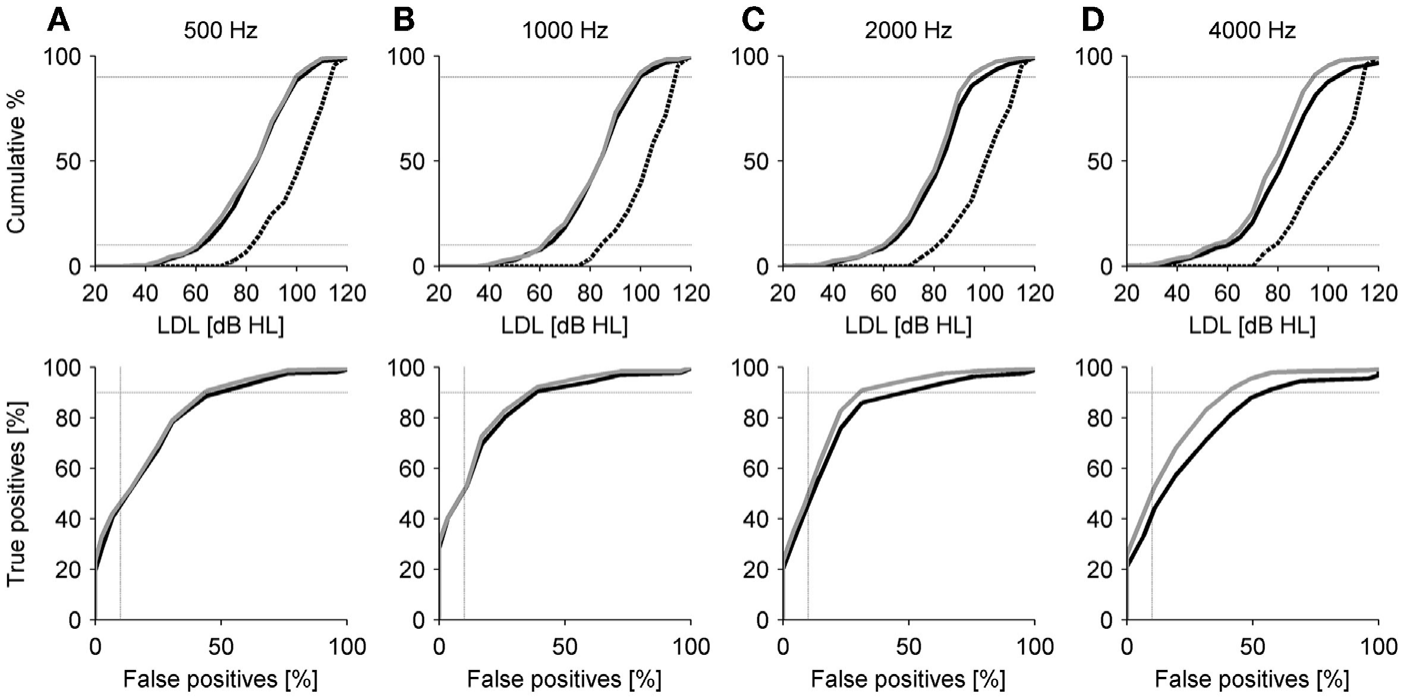
**Comparison of patient LDLs to a normal-hearing reference group (**12). Top panels: Cumulative LDL distributions. Black solid lines – all patients; gray solid lines – patients with normal hearing thresholds; black dashed lines – reference group. Dotted lines indicate 10 and 90% level. Bottom panels: Receiver operating characteristic curves (see Materials and Methods). Black solid lines – all patients compared to reference group; gray solid lines – patients with normal hearing thresholds compared to reference group. Dotted lines indicate 90% true positives and 10% false positives. Panels **(A**–**D)** show the data for 500, 1000, 2000, and 4000 Hz, respectively.

Loudness discomfort level values could also be helpful for clinical assessment of hyperacusis. We therefore compared our patient data to normative LDL values reported by Sherlock and Formby (12), who measured HTs and LDLs of 55 participants with normal hearing and without any known hearing problems. The results are shown in Figure 3. The cumulative distributions of the LDL values of all patients (black line), patients with normal HTs (gray lines) and the reference group (black dashed lines) at 0.5, 1, 2, and 4 kHz are shown in the top panels. The cumulative distribution functions show a very similar shape for the patient and the reference group, with the patient LDL distributions simply shifted to lower sound intensities. Based on the cumulative distributions, we constructed ROC curves (see Materials and Methods) to visualize the discrimination performance that can be achieved with a (purely) LDL-based hyperacusis diagnosis by simply classifying an LDL lower than or equal to a certain threshold as hyperacusis, and a higher LDL as normal. The resulting ROC curves for all possible threshold values are shown in the bottom panels of Figure 3. The horizontal dotted lines at 90% true positives help determine sensitivity, and the vertical dotted lines at 10% false positives serve as a visual aid to assess specificity. This analysis was performed for all patients vs. controls (black lines) and only patients with normal HTs vs. controls (gray lines). The latter analysis was included since the control group had normal hearing as well. The ROC curves show that there is a significant trade-off between detection of hyperacusis and false alarms. To achieve 90% correct classification of the hyperacusis patients, around 40–50% false positives need to be accepted (Figure 3 and Table 2).

**Table 2:** **LDL threshold values to correctly classify at least 90% of hyperacusis patients and associated percentage of false positives**.

	500 Hz	1 kHz	2 kHz	4 kHz
Threshold for ≥90% detection, all hyperacusis patients (dB HL)	105	100	100	105
Associated false positive rate (%)	60.2	39.0	48.3	57.6
Threshold for ≥90% detection, patients with normal hearing (dB HL)	100	100	95	95
Associated false positive rate (%)	44.1	39.0	31.4	41.5

**Figure 4:**
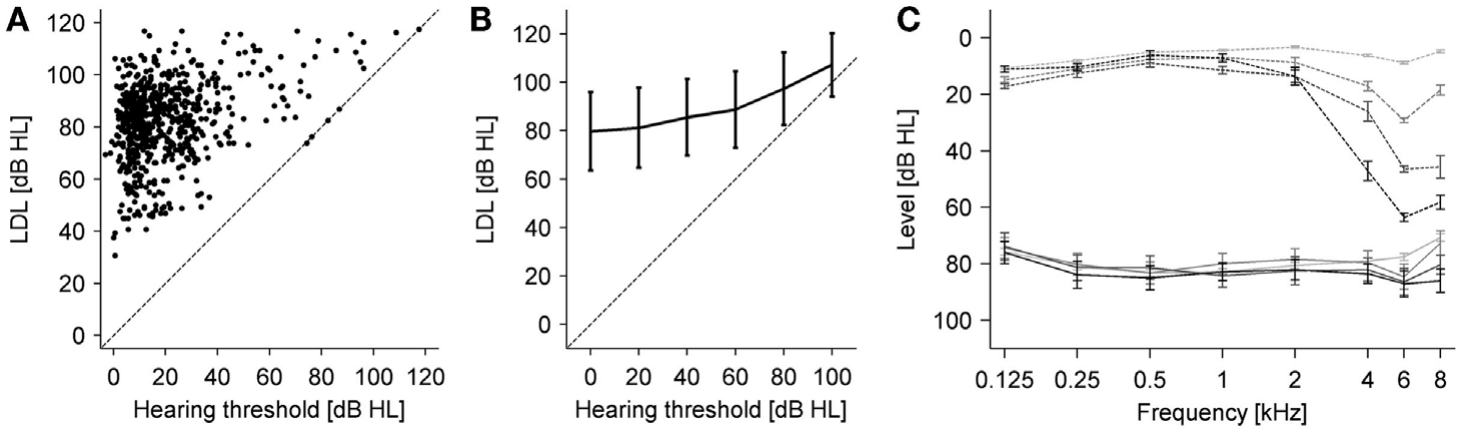
**Relation between LDLs and hearing thresholds**. **(A)** Average LDLs (0.5–6 kHz) vs. average hearing thresholds (0.5–6 kHz) for all participants. There was a significant positive correlation between average hearing thresholds and average LDL (*r* = 0.36, *p* < 0.001). **(B)** Average LDLs for different hearing threshold categories. Hearing thresholds (all patients and all frequencies) were binned using 20 dB bins starting at −10 dB HL, and the average LDL computed for each bin. The dashed line denotes identity. Error bars are ±1 SD. The same relation between hearing thresholds and LDLs was observed in this analysis. Error bars denote ±1 SD. **(C)** Average hearing thresholds (dashed lines) and LDLs (solid lines) of patient groups selected for having different degrees of high-frequency hearing loss (see text). The three hearing loss groups had almost identical LDLs in the hearing loss region, which were slightly elevated compared to the group without hearing loss. Error bars denote ± SEM.

In order to investigate the relation between hyperacusis and hearing loss, we first plotted average LDLs (averaged for each ear across 0.5–6 kHz) against HTs averaged in the same way (Figure 4A). Interestingly, there was no obvious dependence of the LDLs on the HTs besides the fact that the LDL cannot be below the HT, which might also be the main driver for the positive correlation between LDLs and HTs that we found (*r* = 0.36, *p* < 0.01). We analyzed the relation further by grouping individual HT measurements results (regardless of frequency and patients) into 20 dB-wide hearing loss categories, and calculating the corresponding mean LDL for each hearing loss category. This analysis showed a similar positive relation between HTs and LDLs (Figure 4B). Finally, we determined LDLs for four different subgroups of the patients chosen for different degrees of hearing loss. The patients were selected such that all ears had normal HTs up to 2 kHz. At frequencies of 4 kHz and higher they showed different severities of hearing loss. The mean audiograms are depicted with dashed lines in Figure 4C, and the corresponding LDLs with solid lines. With an increase in high-frequency hearing loss, LDLs are also slightly increased in the hearing loss region, albeit to a much smaller degree than the HTs. Moreover, especially the LDL values at 6 kHz were almost identical for the three different degrees of hearing loss, showing that in this case, hearing loss can be ruled out as a determining factor for hyperacusis.

## Discussion

In this study, we have analyzed audiometric data from 381 patients with a primary complaint of hyperacusis. We have found that on average the LDLs are almost flat across frequencies from 125 to 8 kHz, and decreased by about 16–18 dB compared to a reference group (12), indicating a certain generalized, frequency-independent distortion of auditory processing or loudness evaluation. Our findings thus complement and extend the results of previous studies, where the same pattern was reported for the frequency range from 0.5 to 4 kHz (4) or 1 to 8 kHz (13). They are also in good accord with the results of Norena and Chery-Croze (15), who studied loudness growth and found that sounds were rated as uncomfortably loud for roughly the same intensity at all three frequencies that they investigated.

In our data, there was a large spread in the LDL values, which could be as low as 30 dB HL in some patients, whereas for several patients, the LDL was not reached up to the intensity limit of the audiometer. Thus, LDL values might not always adequately reflect subjective perception in hyperacusis, since all patients sought treatment for problems with sound sensitivity. This might be because we measured LDLs using pure-tone stimuli, and the response to pure tones might reflect perception of real-world sounds only to a certain degree. Pure tones are narrow-band whereas real-world sounds usually comprise a broad frequency range, which can also affect how loudly they are perceived at the same sound intensity. Interestingly, when hyperacusis patients were tested with natural sounds like dog barking or baby crying, LDLs were often considerably lower than for pure tones (4), suggesting that natural sounds might be more suitable for quantifying sound sensitivity problems.

By comparing patient LDL values to those of a normal-hearing reference population [data from Sherlock and Formby (12)], we have assessed sensitivity and specificity of LDL measurements for diagnosis of hyperacusis. Particularly low LDL values, e.g., LDLs below 70 dB HL, were highly specific for hyperacusis, but in general LDL values neither showed the required degree of sensitivity nor specificity to serve as a sole diagnostic indication for hyperacusis. In the literature, it has been suggested that LDLs below 100 dB HL might indicate hyperacusis (16), or LDLs below 90 dB HL at least at two frequencies (17). While these values roughly correspond to the threshold values for “detecting” 90% of hyperacusis cases in our data set (Table 2), our result show that this detection performance is associated with a high rate of false positives, indicating that it might be difficult to derive a criterion value for diagnosing hyperacusis. Moreover, LDL measurement results can also depend on the instructions given to the patient and possibly also on the level of trust between clinician and patient. Therefore, LDL measurements can only be one aspect to diagnose hyperacusis, in addition to other symptoms like annoyance, discomfort, and fear of sound, as suggested for example in Andersson et al. (18).

On average, our hyperacusis patients had mild high-frequency hearing loss, but the spread was large, which is similar to the findings of Anari et al. (4). In our patient sample, roughly one-third of the ears tested had HTs within the normal range (i.e., ≤20 dB HL up to 8 kHz), and the remaining patients covered the full range from mild to severe hearing loss. We therefore did not find a specific audiometric pattern associated with hyperacusis, but we can conclude that hearing loss, at least in the form of increases in HTs, is not required for the development of hyperacusis. If hearing loss was required for hyperacusis, one would also expect a negative correlation between HTs and LDLs, but we found the opposite pattern in form of a moderate positive correlation. Therefore, if hyperacusis was initiated by cochlear damage, as one might speculate based on the relation between tinnitus and hyperacusis, it would need to be cochlear damage that does not necessarily affect HTs. Such “hidden hearing loss,” i.e., deafferentation of auditory nerve fibers without permanent HT shifts, has recently been reported for mice and guinea pigs after noise exposure (19, 20), and a recent study has also reported that mice with this kind of cochlear damage show signs of hyperacusis in measurements of acoustic startle (21).

An important difference between patients with a primary complaint of hyperacusis and those with a primary complaint of tinnitus seems to be the fraction of patients with normal HTs. In our sample of hyperacusis patients, 27.5% of the patients had normal HTs in both ears, which is considerably higher than the proportion of normal-hearing tinnitus patients, which is around 7–8% (22, 23). Moreover, the average HTs at 6–8 kHz of a sample of 803 tinnitus patients have been reported to be around 50 dB HL (24), whereas our hyperacusis group only showed HTs of around 30 dB HL at these frequencies, demonstrating clear audiometric differences between tinnitus and hyperacusis patients. It should also be noted that the LDLs of patients with a primary complaint of tinnitus tend to be in the normal range (13, 25). Moreover, the pattern of hyperacusis-related changes in loudness perception in our data seems to be approximately constant across the frequency range, and unrelated to the pattern or degree of hearing loss. This is in stark contrast to tinnitus, where the pitch of the sensation is located in the frequency range of hearing loss (24, 26, 27), and usually narrow-band sounds are described by the patients (28). This discrepancy might be important for evaluating the relation between hyperacusis and tinnitus. The two phenomena are often thought to be related since they often occur together, and it has also been suggested that hyperacusis might be a precursor for tinnitus (6, 29). However, the dissimilarity between the frequency-extent of perceptual distortions and the differences in the degree of hearing loss cast doubt upon hypotheses of a shared mechanism.

Our data suggest that hyperacusis is characterized by a generalized increase in sensitivity or responses to sound across the hearing range. However, the underlying mechanisms that cause this phenomenon remain to be determined. To a certain degree, our audiometric results indicate that hyperacusis might be due to a dysfunction in the central rather than the peripheral auditory system. However, a peripheral factor not captured by our measurements is efferent feedback to the outer hair cells. It is conceivable that a disruption of efferent feedback could contribute to hyperacusis, since the efferent system normally reduces cochlear and auditory nerve responses to loud sounds (30). Measurements of efferent function, for example, through contralateral suppression of opoacoustic emissions (30), were unfortunately beyond the scope of our study. The acoustic reflex thresholds of hyperacusis patients, on the other hand, were found to be in the normal range (4, 14), suggesting that changes in the auditory brainstem nuclei involved in this reflex do not contribute to hyperacusis. This is in contrast to tinnitus, which has been linked to changes in the cochlear nucleus (31, 32).

Recent studies that used earplugs to simulate conductive hearing loss in normal-hearing volunteers for several days have managed to shed some light on putative mechanisms of hyperacusis. After wearing the earplug for several days, the study participants rated sounds as louder than before, especially rating categories like “loud” and “too loud” had shifted by several deicbel (33, 34). Additionally, the majority of subjects also reported hearing phantom sounds (tinnitus) after several days of earplug-induced auditory deprivation (35). All changes were completely reversible, exaggerated loudness reverted to normal within several hours after the earplug was taken out. Interestingly, the changes in loudness occurred also at frequencies where the earplug did not provide much attenuation (33, 34), and even for sounds presented to the ear that had remained open when only one ear was plugged (34). These findings suggest that the evaluation of loudness might occur at a rather high level of the auditory system by pooling across frequencies and ears. Changes in perceived loudness might then be caused by changes of a certain “master gain” in the auditory system, which would cause equal changes across frequencies. Such a mechanism could also account for the LDL pattern observed in hyperacusis patients. Moreover, these studies demonstrate that earplugs and possibly also sound-avoidance behavior might exacerbate hyperacusis.

A recent neuroimaging study has reported increases in sound-evoked neuronal responses in the auditory midbrain, thalamus, and cortex of subjects with hyperacusis (36). Such increases in sound-evoked neuronal activation might reflect increased neuronal response gain, which has been proposed as a putative mechanism for hyperacusis (37). Which factors might lead to such a pathological increase in response gain has so far remained elusive. The fact that a single incident of acoustic shock can lead to long-lasting hyperacusis symptoms (38) indicates that plastic changes in the brain leading to hyperacusis can happen rapidly. Moreover, a recent study in mice indicates that the brain might be more vulnerable to develop hyperacusis during development, since temporary conductive hearing loss in young mice caused hyperacusis-like behavior and even an increased susceptibility to audiogenic seizures that lasted into adulthood (39). How well such animal results translate to humans remains to be determined. We hope that the comprehensive data set that we have presented here will inspire future investigations into the mechanisms of hyperacusis, including the evaluation of potential animal models and theoretical investigations using computer models.

## Conflict of Interest Statement

The authors declare that the research was conducted in the absence of any commercial or financial relationships that could be construed as a potential conflict of interest.
